# The Microbial Detection Array Combined with Random Phi29-Amplification Used as a Diagnostic Tool for Virus Detection in Clinical Samples

**DOI:** 10.1371/journal.pone.0022631

**Published:** 2011-08-10

**Authors:** Lena Erlandsson, Maiken W. Rosenstierne, Kevin McLoughlin, Crystal Jaing, Anders Fomsgaard

**Affiliations:** 1 Virus Research and Development, Department of Virology, Statens Serum Institut, Copenhagen, Denmark; 2 Lawrence Livermore National Laboratory, Livermore, California, United States of America; University of Georgia, United States of America

## Abstract

A common technique used for sensitive and specific diagnostic virus detection in clinical samples is PCR that can identify one or several viruses in one assay. However, a diagnostic microarray containing probes for all human pathogens could replace hundreds of individual PCR-reactions and remove the need for a clear clinical hypothesis regarding a suspected pathogen. We have established such a diagnostic platform for random amplification and subsequent microarray identification of viral pathogens in clinical samples. We show that Phi29 polymerase-amplification of a diverse set of clinical samples generates enough viral material for successful identification by the Microbial Detection Array, demonstrating the potential of the microarray technique for broad-spectrum pathogen detection. We conclude that this method detects both DNA and RNA virus, present in the same sample, as well as differentiates between different virus subtypes. We propose this assay for diagnostic analysis of viruses in clinical samples.

## Introduction

A common method of choice for clinical pathogen identification is polymerase chain reaction (PCR) [1] which is a sensitive and specific method. However, each PCR reaction only provides identification of one specific virus, or a group of related viruses. Thus, a clinical hypothesis regarding the suspected pathogen needs to guide the decision as to which PCR analyses to perform, resulting in a large number of PCR protocols needed to facilitate the identification of all human pathogens.

DNA microarray testing has emerged as a promising new technology for broad-spectrum virus detection, making it possible to test for the presence of thousands of viruses simultaneously. Several microarray platforms have been developed for detection of all known viruses, as well as novel viruses related to known virus families [2], [3], [4]. Some of them have been tested on clinical samples, mostly respiratory samples [2], [3], [4], [5]. A microarray containing probes for all human pathogens could be used for diagnostic analysis, as well as for research purposes, environmental samples or epidemiological surveillance. Moreover, with some clinical syndromes or symptoms, it might be difficult to determine which viruses to test for in order to establish the correct diagnosis since many viruses can present similar symptoms. For this reason various multiplexed assays with virus panels, utilizing PCR or other methods, have been established. However, a microarray could be viewed as an extended pathogen panel, covering all viruses and bacteria, increasing the chance of the correct identification of one or several pathogens even in situations without any prior knowledge or suspicion about the etiology.

Without a pre-amplification of the clinical sample, such as PCR or virus culture, most microarrays are not sensitive enough for pathogen identification in clinical samples since virus titres normally are below their detection limit. For example, a diagnostic human Papilloma virus (HPV)-specific microarray utilizes an HPV-specific PCR-amplification prior to microarray hybridization [6], [7]. Different PCR-based random amplifications have been developed to achieve enough material for a successful identification [3], [4], [8]. However, studies have shown that PCR-based whole genome amplification (WGA) may generate incomplete coverage of genomes [9]. Instead we used multiple displacement amplification (MDA)-based WGA through isothermal amplification by the Phi29 polymerase [9]. The Phi29 polymerase has the highest processivity rate reported, ∼70,000 bases every time it binds [10] and an error rate of only 1 in 10^6^–10^7^ bases [11], allowing for good coverage across genomes [9], [12], [13]. The average length produced is >10 kb compared to ≤3 kb by the Taq polymerase [9]. Phi29-amplification prior to microarray analysis has been used for human genomic DNA analysis [14], [15], [16] and for bacterial DNA analysis [17], [18]. However, the Phi29 polymerase cannot amplify RNA or short DNA fragments (<2000 bp), such as cDNA generated with random hexamers. To overcome this, the method Whole Transcriptome Amplification (WTA) has included a ligation step prior to amplification [2] resulting in cDNA fragments that are ligated into larger transcriptomes that can be efficiently amplified by the Phi29 polymerase.

Here we investigate real life utility and sensitivity of the microarray technique in a diagnostic set-up. We have established a protocol including pre-treatment, nucleic acid (NA) purification and Phi29-amplification followed by microarray analysis of DNA and RNA viruses from a diverse set of clinical samples. We show that Phi29-amplification generates enough material for successful identification by the Lawrence Livermore Microbial Detection Array (LLMDA) [19], [20], demonstrating the potential of the microarray technique for broad-spectrum pathogen detection in clinical samples.

## Materials and Methods

### Ethics statement

Exemption for review by the ethical committee system and for obtaining informed consent was obtained from the Committee on Biomedical Research Ethics for the Capital region in accordance with Danish law on quality control and assay development projects.

### Virus and clinical samples

This study used clinical samples already received for routine diagnostic analysis at the Department of Virology, Statens Serum Institut (SSI), Copenhagen, Denmark (Danish National reference laboratory (ISO 17025;2005); www.ssi.dk), containing DNA or RNA viruses. DNA viral genomes were linear or circular double-stranded: Herpes Simplex virus (HSV) 1 and HSV2, BK Polyomavirus (BKV), JC Polyomavirus (JCV) and HPV subtypes 6, 16, 53, 61. RNA viral genomes were (i) segmented double-stranded: Rotavirus A (Rota A, 11 segments), (ii) (+)single-stranded: Astrovirus, Sapovirus, Dengue 1, Hepatitis C virus (HCV) and Enterovirus (EV), and (iii) (−)single-stranded: human Respiratory Syncytial virus (RSV). Clinical samples were cerebrospinal fluid (CSF), urine, serum, cervical smear, faeces, skin lesion and tracheal aspirate (TA). Techniques used for routine diagnostic analysis of viruses were in-house real-time PCR assays (ISO 17025;2005) for HSV1, HSV2, BKV, JCV, Rota A, Astrovirus, Dengue, EV and RSV, previously published assay for HCV [21] and Sapovirus [22], and Clart® HPV2 microarray (Genomica) for HPV. PCR's were performed on Mx3005P (Stratagene) or ABI7900 (Applied Biosystems) thermal cyclers.

### Pre-treatment and extraction of clinical samples

Five- to two-hundred microliters of clinical sample was centrifuged at 17,000 g for 10 minutes. The supernatant was filtered through a 0.22 µm Ultrafree MC spin filter (Millipore) at 2000 g for 2 minutes and DNase treated (6 U DNaseI Amplification grade, Invitrogen) for 1½ hours at room temperature while shaken in a Thermomixer (AH Diagnostics). The viral NA was extracted using the PureLink Viral RNA/DNA kit (Invitrogen), without the addition of carrier RNA. The extracted viral NA was eluted with 20–30 µl DNase/RNase-free sterile water, and stored at −20°C or immediately used.

### Reverse transcription

Reverse transcription on purified viral RNA was performed using the Superscript VILO cDNA synthesis kit (Invitrogen) according to protocol. Briefly, 14 µl of extracted viral RNA was put into a 20 µl-reaction containing Superscript enzyme and random primers, and incubated at 42°C for 1 hour. The enzyme was inactivated at 95°C for 5 minutes and the sample stored at −20°C or immediately used.

### Phi29 polymerase amplification

Viral DNA was amplified by Phi29 polymerase amplification using GenomiPhi V2 Amplification kit (GE Healthcare) or Repli-g Midi kit (typical yield ∼40 µg from a 50 µl reaction, Qiagen), following manufacturer's protocols. Briefly, using GenomiPhi V2 Amplification kit, 1 µl of purified DNA was added to 9 µl of sample buffer, incubated at 95°C for 3 minutes and cooled on ice. Ten microliters of GenomiPhi reaction was added, containing Phi29 polymerase and random primers, and the reaction incubated at 30°C for 1 ½ hours. The reaction was terminated by incubation at 65°C for 10 minutes. The amplified viral DNA was stored at −80°C or immediately used. Briefly, using Repli-g Midi kit, 5 µl of purified DNA was mixed with 5 µl denaturation buffer, incubated at room temperature for 3 minutes and the reaction terminated by the addition of 10 µl stop solution. Thirty microliters of Repli-g reaction mix, containing Phi29 and random primers, was added and the reaction incubated at 30°C for 16 hours. The reaction was terminated by incubation at 65°C for 3 minutes. Amplified material was purified using QIAamp DNA mini kit (Qiagen) using a modified protocol (www.qiagen.com), and checked for purity and concentration using a NanoDrop spectrophotometer (Thermo Scientific) and agarose gel electrophoresis. The DNA was stored at −80°C or immediately used for microarray analysis.

### Whole transcriptome amplification (WTA)

For viral NA amplification (both DNA and RNA virus) we used the described WTA method [2] by using the QuantiTect Whole Transcriptome Amplification kit (typical yield ∼40 µg from a 50 µl reaction, Qiagen) according to manufacturer's protocol, except for the reverse transcription step that was replaced by the Superscript VILO kit (Invitrogen) described above. The WTA protocol includes 3 sequential reactions: first a reverse transcription reaction to generate cDNA, secondly a ligation of generated cDNA into larger transcriptomes and thirdly a Phi29-amplification of the generated transcriptomes. Briefly, as described above, 14 µl of extracted viral NA was put into a 20 µl-VILO reaction and incubated at 42°C for 1 hour. The Superscript enzyme was inactivated at 95°C for 5 minutes, 10 µl of the VILO cDNA reaction was added to 10 µl of ligation mix and incubated at 22°C for 2 hours. Thirty microliters of amplification mix containing Phi29 polymerase and random primers was then added and amplification performed at 30°C for 8 hours. The reaction was terminated by incubation at 95°C for 5 minutes. Amplified material was purified using QIAamp DNA mini kit (Qiagen) using a modified protocol (www.qiagen.com), and checked for purity and concentration using a NanoDrop spectrophotometer (Thermo Scientific) and agarose gel electrophoresis. The DNA was stored at −80°C or immediately used for microarray analysis.

### Quantification and conformation by real-time PCR

For the quantification of virus before and after Phi29-amplification, virus-specific real-time PCR's were performed. These were in-house assays for HSV1, HSV2, HPV6, HPV16, BKV, Rota A, Astrovirus, EV and RSV. For the detection of human gDNA, an in-house assay for β-actin was used (95°C for 15 minutes, 40 cycles of 95°C for 15 seconds and 55°C for 1 minute; Forward primer: CTC TTC CAG CCT TCC TTC CT, Reverse primer: AGC ACT GTG TTG GCG TAC AG, Probe: Yakima Yellow-TGG AGT CCT GTG GCA TCC ACG A-BHQ. In addition, previously published assays were used for JCV [23], HCV [21] and Sapovirus [22]. For the multi-HPV (6, 16, 53, 61) positive sample (determined in the routine by Clart® HPV2 microarray, Genomica), only HPV 6- and 16-specific PCR's were performed before and after Phi29-amplification. For the quantitative analysis of BKV, we used an in-house standard containing 20,000–2×10^7^ copies/ml. For the confirmation of additional viruses found by microarray analysis, in-house real-time PCR's were used for HPV103, Picobirnavirus, Rota C and Dengue subtypes (1, 2, 3 and 4), and previously published assays for JCV [23], GB virus type C (GBV-C) [24] and Human Adenovirus C (HAdV-C) [25]. Thermal cycling was performed in a thermal cycler (Mx3005P (Stratagene) or ABI7900 (Applied Biosystems)). Fold difference was calculated from ΔCt obtained from real-time PCR results before and after Phi29-amplification, combined with dilution factors for each sample. In doing so, we also made the assumption that a change in Ct-value was equivalent to a doubling of target DNA.

### Microarray analysis

 The microarray used was the LLMDA version 2, developed at Lawrence Livermore National Laboratory (LLNL), USA and already described elsewhere [19], [20]. Briefly, the LLMDA contains 388,000 probes designed from all sequenced virus and bacteria, resulting in a large number of probes covering each genome. The microarray was designed for both detection and discovery, to both identify known organisms and to detect un-sequenced or emerging organisms, while strain or subtype identification was not a goal. The array have both conserved and unique probes within each family, where oligos that were conserved, to the extent possible, within a family, and unique relative to other families and kingdoms, were prioritized [19]. Labelling and microarray hybridization was performed at NimbleGen Systems-Iceland, Iceland, or in-house at SSI, Denmark according to manufacturer's protocol (Gene expression analysis, Roche NimbleGen). Briefly, 1 µg of heat-denatured WTA-amplified sample was labelled using nick translation with Cy3-labeled random nonamer primers (TriLink Biotechnologies) and Klenow fragment (exo-) (NEB) at 37°C for 2 hours. Labelled DNA was iso-propanol precipitated and the pellet washed, dried, reconstituted and quantitated. For hybridization, the in-house assay used 12 µg of labelled DNA whereas NimbleGen Systems-Iceland used 6 µg. Hybridization was performed in a NimbleGen Hybridization system using the NimbleGen Hybridization kit (Roche NimbleGen).

Microarrays were scanned using a GenePix 4000B Scanner (Molecular Devices) and data analysed using the maximum likelihood method developed at LLNL, extensively described elsewhere [19]. Additional stringency criteria were applied to exclude bacterial sequences and viruses having fewer than 20% of probes detected out of those expected. Briefly, probes were classified as detected if the intensity exceeded a threshold equal to the 99^th^ percentile of intensities for negative control probes, except that one sample was analysed using the 95^th^ percentile. Targets in an internal database of 43 705 viral sequences, developed at LLNL [19], were screened against the stringency criteria; an unconditional log-odds score for presence of each remaining target was computed, and targets having log-odds scores less than 5 were excluded. A greedy forward selection algorithm was applied to find the collection of targets most likely to be present in the sample. At every forward selection step, a conditional log-odds score was computed for each remaining target, representing its potential contribution to the log-likelihood conditioned on the presence of the previously selected targets; the target having the largest conditional log-odds was selected and added to the collection. The resulting conditional and unconditional log-odds scores were plotted in a bar graph format [19], where the conditional log-odds scores shows the contribution from a target that cannot be explained by another, more likely target above it, and the unconditional score illustrates that some very similar targets share a number of probes, so that multiple targets may be consistent with the hybridization signals.

As a complement, another mode of data analysis was developed at SSI, where data was processed using the NimbleScan software program (NimbleGen, Roche). A mean of intensities for each probe-set was calculated. Outlier intensities within each probe-set were filtered out when signals were more than 3 standard deviations from the mean of the probe-set. A signal threshold was defined as the 99^th^ percentile of the random control intensities, and every probe-set mean containing more than 10 signals above this threshold was considered as a positive signal.

Microarray data has been submitted to the Gene Expression Omnibus (GEO) database http://ncbi.nlm.nih.gov/geo/ with the accession number GSE28597. All microarray data used in this study is MIAME compliant.

## Results

### Sample pre-treatment and Phi29-amplification

**Figure 1:**
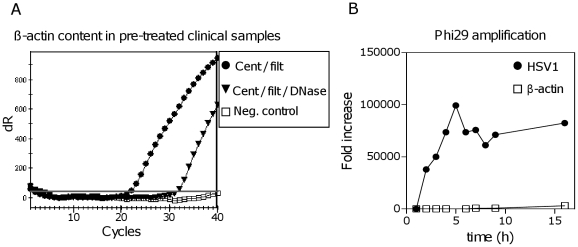
The human genomic DNA levels were reduced by pre-treatment. Comparing the effects of different pre-treatment methods on the level of human gDNA (measured as ß-actin), in a clinical sample. A HSV1^+^ skin lesion-sample was pre-treated with centrifugation and filtration only, or with centrifugation, filtration and DNase treatment. The ß-actin content was measured by real-time PCR. (A) Centrifugation+filtration: Ct = 22; centrifugation+filtration+DNase treatment: Ct = 32; ΔCt = 10 which equals a 1000-fold decrease in signal. Negative control was water used as a PCR control. (B) Time-course for Phi29-amplification of pre-treated clinical sample (centrifugation+filtration+DNase) using GenomiPhi (GE Healthcare). Fold increase in signal plotted versus amplification time for HSV1 and ß-actin.

To remove contaminating human gDNA present in clinical samples, we designed a pre-treatment protocol including centrifugation, filtration and DNase treatment. It has been demonstrated that filtration, DNase and RNase treatment can increase the efficiency of downstream amplifications [26], [27]. During DNase treatment the viral NA should be protected inside the viral particle [28]. After pre-treatment, the viral particles were lysed, viral NA purified and later Phi29-amplified using GenomiPhi V2 Amplification kit (GE Healthcare). During purification, the addition of carrier RNA did not improve the yield and interfered with the downstream Phi29-amplification, and was therefore left out (data not shown). Quantitative analysis of viral DNA or human gDNA before and after pre-treatment or Phi29-amplification was done by real-time PCR (Figure 1 and Table S1). Only centrifugation and filtration left large amounts of human gDNA still present in a HSV1^+^ sample (Ct = 22), while DNase treatment reduced the ß-actin signal 1000-fold (Ct = 32) (Figure 1A). The DNase treatment also had an effect on the HSV1, resulting in a 3- to 20-fold decrease in virus signal after DNase treatment (Table S1). However, this was accompanied by a much larger fold increase after Phi29-amplification compared to without DNase treatment (e.g. 88,292 compared to 1176), resulting in lower Ct-values and more viral DNA available for downstream analysis (Table S1). This was also seen for the RNA virus HCV (Table S1) as well as for other DNA and RNA viruses (data not shown). To investigate to what extent Phi29-amplification of remaining human gDNA would occur we performed a time-course experiment with the amplification-time extended up to 16 hours (Figure 1B). The ß-actin signal was still at acceptable background level (3000-fold increase in signal), while the HSV1 signal showed an 80,000–100,000-fold increase in signal. In conclusion, our pre-treatment removed a significant amount of human gDNA, which allowed for good virus amplification.

### Whole transcriptome amplification

**Figure 2:**
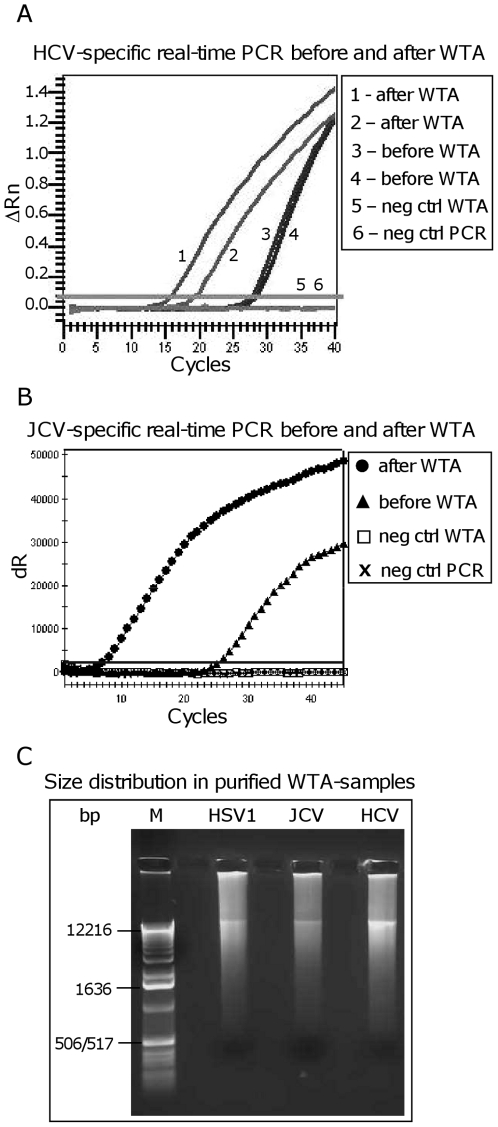
The WTA amplification method amplifies both DNA and RNA viruses. The described WTA amplification method was used to successfully amplify DNA or RNA viruses, present in clinical samples. Negative control for both WTA and PCR was water. (A) Analysis of RNA virus. A HCV-specific real-time PCR was used on a HCV^+^ serum sample, before and after WTA. Duplicate samples. (B) Analysis of DNA virus. JCV-specific real-time PCR on a JCV^+^ CSF sample, before (undiluted) and after WTA (diluted 20-fold). (C) Purified WTA samples (HSV1, JCV and HCV) are run on a 0.8% agarose gel to analyse size distribution, and 500 ng per sample was loaded. Marker is TrackIt 1 Kb Ladder (Invitrogen) ranging from 500 bp to 12 kb.

To establish an amplification protocol suitable for both DNA and RNA viruses, we used the described Phi29-amplification method WTA [2] that has included a ligation step prior to the amplification step, thereby generating larger templates for Phi29. The WTA performed on a HCV^+^ serum sample (RNA virus) resulted in a ∼200,000-fold increase in signal (Figure 2A and Table 1), as compared to only 3-fold without ligation (data not shown). The WTA RT-reaction and ligation step did not have any adverse effects when performed on a DNA-virus such as JCV from a CSF sample (Figure 2B and Table 1), enabling us to run both viral DNA and RNA through the same amplification protocol prior to microarray hybridization. The WTA was used on 12 different DNA or RNA viruses found in 7 different types of clinical samples (Table 1 and Figure 2). The fold increase in PCR signal comparing before and after WTA, ranged from 330 to 564×10^6^. The highest fold increase was detected for the circular viruses JCV, BKV and HPV (Table 1 and Figure 2B). Such circular genomes have previously been shown to be efficiently amplified by Phi29 polymerase, through rolling circle amplification (RCA) [29], [30]. The lowest increase in signal was seen for Rota A found in faeces (Table 1) due to large quantities present already before amplification (Ct = 12), which resulted in a Ct-value of 7 after WTA amplification (Table 1). The fold increase in PCR signal for gDNA (β-actin) was analysed in samples representing all 7 types of clinical samples, and showed no or low levels of β-actin amplification (Table S2). WTA-amplified samples were purified and typical yield after 8 hours of WTA was ∼25 µg (data not shown) showing good size distribution (Figure 2C).

**Table 1:** The WTA method performed on a diverse set of clinical samples.

Group^a^	Viral family	Virus	Size	Sample	ΔCt^b^	Fold increase^c^
**dsDNA**	Herpesviridae	HSV1	152 kb, lin	lesion	27-13	305,000
	Herpesviridae	HSV2	155 kb, lin	lesion	27-15	185,000
	Papillomaviridae	HPV16	7.9 kb, cir	Cervix	26-18	24,000
	Papillomaviridae	HPV6,16,53,61^d^	7.9 kb, cir	Cervix(6)	35-12	14×10^6^
				Cervix(16)	35-16	250×10^6^
	Polyomaviridae	BKV	5.1 kb, cir	Urine	37-16	133×10^6^
	Polyomaviridae	JCV	5.1 kb, cir	CSF	27-7	564×10^6^
**dsRNA**	Reoviridae	Rota A	17.4 kb, seg	Faeces	12-4	330
**(+)ssRNA**	Astroviridae	Astrovirus	6.5 kb	Faeces	19-10	1900
	Caliciviridae	Sapovirus	7.5 kb	Faeces^e^	26-18	5800
				Faeces^f^	21-11	6100
	Flaviviridae	Dengue 1	10.7 kb	Serum	n.a.	n.a.
	Flaviviridae	HCV	9.6 kb	Serum	29-17	207,000
	Picornaviridae	EV	7.4 kb	Faeces	19-6	94,000
**(−)ssRNA**	Paramyxoviridae	RSV	15.2 kb	TA	27-18	11,500

**NOTE**. WTA, Whole Transciptome Amplification; HSV1, Herpes Simplex virus 1; HSV2, Herpes Simplex virus 2; HPV, Human Papillomavirus; BKV, BK Polyomavirus; JCV, JC Polyomavirus; CSF, cerebrospinal fluid; Rota A, Rotavirus A; HCV, Hepatitis C virus; EV, Enterovirus; RSV, Respiratory Syncytial virus; TA, tracheal aspirate; n.a., not analysed.

a Viruses are grouped based on nucleic acid content, according to the Baltimore Classification.

b Difference in Ct-value in virus-specific real-time PCR before and after Phi29-amplification.

c Fold increase of virus after Phi29-amplification, calculated from ΔCt combined with dilution factors for each sample.

d Only HPV6- and HPV16-specific real-time PCR's were performed.

e Sapovirus from a double-positive Astrovirus/Sapovirus faeces sample.

f Sapovirus from a single-positive Sapovirus faeces sample.

### Microarray analysis of clinical samples

**Figure 3:**
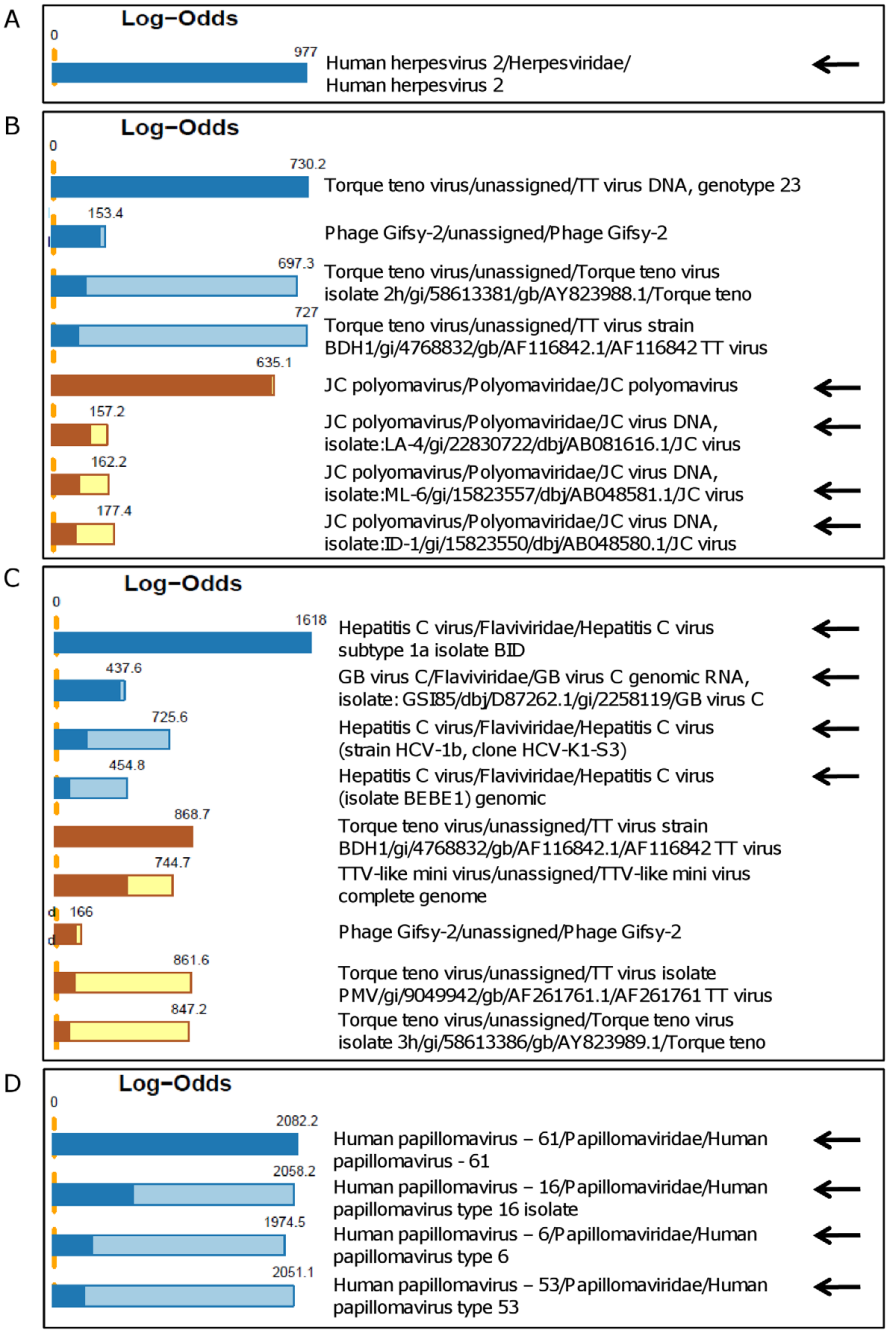
Microarray analysis performed on WTA-amplified clinical samples. Results from the microarray data analysis of WTA-amplified clinical samples, using the maximum likelihood method developed at LLNL [19], with additional stringency criteria applied. The lighter and darker-coloured portions of the bars represent the unconditional and conditional log-odds scores, respectively. The conditional log-odds scores shows the contribution from a target that cannot be explained by another, more likely target above it, while the unconditional score illustrates that some very similar targets share a number of probes. (A) Detection of HSV2 in a HSV2^+^ skin lesion-sample. (B) Detection of JCV and TTV in a JCV^+^ CSF sample. (C) Detection of HCV, GBV-C, TTV and TTV-like mini virus in a HCV^+^ serum sample. (D) Detection of 4 different HPV subtypes (6, 16, 53 and 61) in a multi-HPV^+^ cervical smear sample.

Samples amplified by WTA representing 7 different types of clinical samples were labelled and hybridized to the LLMDA (Figure 3 and Table 2). Microarray data was analysed using the maximum likelihood method developed at LLNL [19], [20], with additional stringencies applied. In 14 out of 14 clinical samples, the expected virus was detected: HSV1, HSV2, HPV16, HPV6/16/53/61, BKV, JCV, Rota A, Astrovirus, Sapovirus, Dengue 1, HCV in duplicate, EV and RSV (Table 2). For the RSV^+^ sample, a detection threshold equal to the 95^th^ percentile of control probes had to be used for detection. For this sample we also used the data analysis developed at SSI, where RSV was detected using a detection threshold equal to the 99^th^ percentile of random control probes (data not shown). Moreover, using this approach on all samples, we generated the same result as seen with the maximum likelihood method (data not shown). For the double-positive faeces sample containing both Astro- and Sapovirus, only Astrovirus was detected by the microarray analysis. In the negative control no viruses were detected.

**Table 2:** Microarray analysis performed on a diverse set of clinical samples.

Clinical sample	Type	Detected viruses	Log-Odds Score	Probe hybridization
**HSV1+**	Skin lesion	**HSV1**	1090.4	172/222
		TTV-like mini	335.2	59/107
**HSV2+**	Skin lesion	**HSV2**	977.0	171/228
**HPV16+**	Cervix	**HPV16**	816.6	148/374
		**HPV103**	541.8	58/90
		TTV	674.4	103/113
**HPV16+53+6+61+**	Cervix	**HPV6**	1974.5	231/331
		**HPV16**	2058.2	248/375
		**HPV53**	2051.1	216/294
		**HPV61**	2082.2	231/295
**BKV+**	Urine	**BKV**	598.4	105/121
		TTV-like mini	359.7	65/107
		JCV	91.0	5/9
**JCV+**	CSF	**JCV**	635.1	103/125
		TTV	730.2	110/116
**Rota A+**	Faeces	**Rota A**	3351.9	498/696
		**Picobirnavirus**	677.5	49/80
		Rota C	284.6	35/39
**Astrovirus+Sapovirus+**	Faeces	**Astrovirus**	707.9	105/120
		TTV	700.3	104/113
**Sapovirus+**	Faeces	**Sapovirus**	301.0	49/141
		**HAdV-C**	675.0	173/682
		TTV	858.0	199/214
		TTV midi	698.7	167/177
		TTV-like mini	699.4	175/235
**Dengue 1+**	Serum	**Dengue type 1**	719.6	92/109
		Dengue type 2	403.3	59/110
		Human endogenous retrovirus	333.2	26/28
**HCV+**	Serum	**HCV**	1618.0	263/268
		**GBV-C**	437.6	60/76
		TTV	868.7	111/112
		TTV-like mini	744.7	86/98
**HCV+**	Serum	**HCV**	1644.4	260/268
		**GBV-C**	396.8	49/76
		TTV	940.2	111/112
		TTV-like mini	799.8	85/98
**EV+**	Faeces	**EV-A (Coxsackie A6)**	1896.1	378/732
		TTV midi	638.3	145/179
		TTV	320.5	88/178
**RSV+**	TA	**RSV**	979.1	69/148
		TTV	849.0	106/112
		TTV midi	651.9	66/80
		TTV-like mini	615.3	63/84
		Human endogenous retrovirus	175.3	9/19
**Neg control**	water	n.d.	-	-

**NOTE**. HSV1, Herpes Simplex virus 1; TTV, Torque Teno Virus; HSV2, Herpes Simplex virus 2; HPV, Human Papillomavirus; BKV, BK Polyomavirus; JCV, JC Polyomavirus; CSF, cerebrospinal fluid; Rota A, Rotavirus A; HAdV-C, Human Adenovirus C; HCV, Hepatitis C virus; GBV-C, GB virus type C; EV, Enterovirus; RSV, Respiratory Syncytial virus; TA, tracheal aspirate; n.d., none detected. Bold represents viruses identified by both microarray and PCR.

In 10 out of 13 clinical samples (77%) representing all 7 types of clinical samples used in the study (skin lesion, urine, CSF, cervical smear, serum, faeces and TA), viruses from the Anelloviridae family were found (Torque Teno virus (TTV), Torque Teno Midi virus (TTMV) and TTV-like mini virus) (Table 2). They were detected in samples containing DNA or RNA viruses, demonstrating that our pre-treatment and amplification protocol followed by microarray analysis can easily detect both types in one sample. Additional viruses were detected in 9 samples (Table 2 and data not shown), with log-odds scores ranging from 91.0 to 677.5. Some were clinically irrelevant since they were phages (Propionibacterium phage, Phage Gifsy-2, Lactococcus phage, Enterobacteria phage ST104, Mycobacteriophage and Pseudomonas phage) or plant viruses (Pepino mosaic virus), probably from digested or environmental sources (data not shown). Human endogenous retrovirus was detected in the Dengue 1 and the RSV sample (Table 2). The rest of the additional viruses found (HPV103, Picobirnavirus, HAdV-C, GBV-C, JCV, Rota C and Dengue 2) were tested with virus-specific PCR for confirmation (Table 2 and data not shown). The HPV103 (cir dsDNA) detected in the HPV16^+^ sample is not present on the microarray used for routine HPV diagnosis (Genomica). Instead its presence was confirmed using a HPV103-specific PCR. The Rota A^+^ faeces sample was found to be positive for Picobirnavirus (dsRNA) and negative for Rota C (dsRNA) by PCR. The Sapovirus^+^ faeces sample was found to be positive for HAdV-C (linear dsDNA) in the microarray analysis, which was confirmed by PCR. Two separate microarray experiments detected GBV-C ((+)ssRNA) in the HCV^+^ sample, which was confirmed by a GBV-C-specific PCR. The BKV^+^ sample was detected as positive for JCV by microarray but tested negative by PCR. The Dengue 1^+^ sample was found to be positive for both Dengue-1 and Dengue 2 in the microarray analysis, but tested negative for types 2, 3 and 4 using Dengue subtype-specific PCR's.

### Microarray detection range for BKV^+^ urine samples

**Figure 4:**
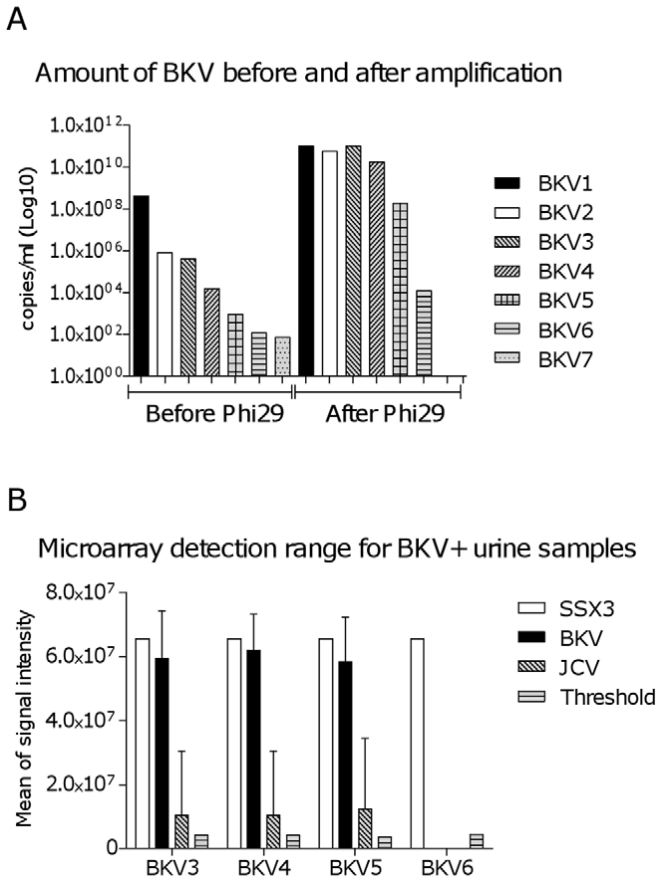
Microarray detection range for Phi29-amplified BKV^+^ clinical samples. Urine samples containing various amounts of BKV, ranging from 4.0×10^8^ to 73 copies/ml (BKV1–BKV7), were Phi29-amplified. Real-time PCR analysis using an in-house BKV-standard determined the number of BKV copies/ml before and after amplification. Selected samples were analysed by microarray. (A) Amount (copies/ml) of BKV before and after Phi29-amplification. (B) Microarray analysis on 4 selected amplified samples (BKV3–BKV6) containing various amounts of BKV. The human genomic sequence SSX3 (synovial sarcoma, X breakpoint 3) was used as a hybridization control.

To investigate the detection range of the microarray on clinical samples, we performed analysis on urine samples positive for the circular virus BKV (Table 3 and Figure 4). Samples ranging from 15,810 to 4.0×10^8^ copies/ml of BKV were amplified by Phi29 polymerase for 16 hours using Repli-g Midi kit (Qiagen). Also included were three dilutions of a BKV urine sample, containing 892, 119 or 73 copies/ml, respectively. Samples containing ≥1000 copies/ml of BKV (BKV1-BKV5) were efficiently amplified with yields from 1.0×10^11^ to 1.9×10^8^ copies/ml of BKV (Table 3 and Figure 4A), and with ∼25 µg of DNA generated after amplification (data not shown). The total input of viral copies per 50 µl Phi29-reaction for BKV1-BKV5, were 2×10^6^, 4100, 2100, 80 and 5 genomic copies, respectively (Table 3). Amplification of samples containing ∼100 copies/ml (BKV6 and BKV7) resulted in loss of reproducibility and low yields (Table 3 and Figure 4A). The total input of viral copies per Phi29-reaction for these two samples were 0.6 and 0.4, respectively (Table 3). Theoretically, amplification of samples containing 200 copies/ml means that ∼1 viral copy is added to the Phi29-amplification reaction, resulting in stochastic problems due to unequal distribution in highly diluted solutions. Four amplifications were selected for microarray analysis (BKV3, BKV4, BKV5 and BKV6) and hybridized to the LLMDA (Table 3 and Figure 4B), and analysed using the data analysis method developed at SSI. BKV^+^ urine samples containing ≥1000 copies/ml were clearly detected by the microarray analysis after Phi29-amplification (Figure 4B). The human genomic sequence SSX3 (synovial sarcoma, X breakpoint 3) was used as a hybridization control and had the same signal intensity in all 4 microarrays. Some cross-hybridization with JCV was seen in the 3 samples where BKV was detected, even if they all were negative in a JCV-specific PCR (data not shown). This is similar to the BKV sample presented in table 2. From this we conclude that, from as little as 5 copies input (≥1000 copies/ml) of BKV genomes the Phi29-amplification was able to generate enough material for a clear detection by microarray analysis.

**Table 3:** Microarray detection range for BKV^+^ urine samples.

Sample	Before Phi29^a^	After Phi29^a^	Input per Phi29-reaction^b^	Detected by LLMDA
**BKV1**	4×10^8^	1×10^11^	2×10^6^	-
**BKV2**	819,400	5.7×10^10^	4100	-
**BKV3**	419,700	1×10^11^	2100	Yes
**BKV4**	15,810	1.7×10^10^	80	Yes
**BKV5**	892	1.9×10^8^	5	Yes
**BKV6**	119	11 910	0.6	No
**BKV7**	73	0	0.4	-

**Note.**

a Copies/ml.

b Number of copies per 5 µl.

## Discussion

By using WTA for viral amplification, both DNA and RNA viruses could be run through the same protocol. The correct DNA or RNA virus was detected in 14 out of 14 samples, representing a diverse set of clinical materials. Moreover, we can correctly detect multiple subtypes of a virus present in a sample, as seen for two clinical samples positive for multiple HPV subtypes (6/16/53/61 and 16/103, respectively), with no cross-hybridization towards other subtypes. Furthermore, EV-A (Coxsackievirus A6) was clearly detected without any cross-hybridization towards any other of the 6 species with several serotypes included in the large Enterovirus genus (EV-B/C/D and Rhinovirus A/B/C). It should be noted that subtype identification was not a goal in the probe design when developing LLMDA, nevertheless, the ability to combine the signals from multiple probes in the analysis made it possible to discriminate between different subtypes [19]. In several samples analysed, more than one virus was found, in some cases including both DNA and RNA viruses. Taken together, this demonstrates the potential and real life utility of the microarray technique for broad-spectrum pathogen detection in clinical samples.

In 4 samples we found viruses not previously tested for in the routine analysis, HPV103 in a HPV16^+^ cervical smear sample, Picobirnavirus in a Rota A^+^ faeces sample, HAdV-C in a Sapovirus^+^ faeces sample and GBV-C in a HCV^+^ serum sample. Two of these were viruses within the same family as tested for before, HPV103 and GBV-C, while the other two were from different families, Picobirnavirus and HAdV-C. Picobirnavirus is a dsRNA virus (Picobirnaviridae) found together with the dsRNA virus Rota A (Reoviridae), while HAdV-C is a dsDNA virus (Adenoviridae) found together with the (+)ssRNA virus Sapovirus (Caliciviridae).

There was a false detection of virus in 3 samples, but they were all within the same family as previously tested for in the routine analysis, JCV in a BKV^+^ urine sample, Rota C in a Rota A^+^ faeces sample and Dengue 2 in a Dengue 1^+^ serum sample. They could all be explained by cross-reactivity between the probes targeting one species with other species in the same viral family. Future improvements to the current probe design can address cross-reactivity issues such as these described. Regarding the failure to detect Sapovirus in the double-positive Astrovirus^+^/Sapovirus^+^ faeces sample, we believe the reason was poor amplification due to low viral copy number rather than a specificity problem, since Sapovirus was detected in the Sapovirus^+^ faeces sample (Table 2). Supporting this is the Sapovirus-specific PCR used before and after Phi29-amplification (Table 1), which indicates that the single-positive sample had more amplified Sapovirus (Ct = 11) after amplification, compared to the double-positive sample (Ct = 18) and thereby a sufficient amount for a successful detection by microarray analysis.

By microarray analysis, TTV and related viruses were detected in 77% of the samples, from 7 different types of clinical samples. We believe this is the first report regarding the prevalence of TTV and related viruses in clinical samples from Denmark. These TTV viruses are reported to frequently infect humans, with as much as 100% in certain populations, but no direct evidence links them to any specific clinical disease [31]. They have circular genomes (cir ssDNA) and are efficiently amplified by the Phi29 polymerase through RCA.

A microarray such as the LLMDA [19], [20] could be a useful tool in a number of ways. Since it enables simultaneous detection of viruses as well as bacteria, it could be useful in e.g. diagnosis of sexually transmitted diseases, or of respiratory illnesses with combined viral and bacterial components. Furthermore, many viruses cause symptoms that clinically are very similar, making it hard to choose the correct diagnostic analysis. In cases of clinical hypothesis failure, the microarray could be a useful tool for finding an etiologic agent in samples that are presumed negative or in samples with unknown etiology. Microarray could also play a dual role combining diagnostics and research, by being a suitable research tool for finding new pathogens. It could also be of great value for epidemiological surveillance, where clinically unimportant viruses or viruses with unknown consequence for the carrier (e.g. TTV, retroviruses or others) could be found, described and followed in both human and animal populations.

The protocol established here enabled us to get proof of concept that microarray analysis can be used to correctly identify viruses present in a diverse set of clinical samples. However, the protocol is at the moment to time and labour intensive to be used in a routine set up. We are currently focusing on reducing the overall assay time and the costs to develop a more suitable protocol for routine usage. Incubation times throughout the protocol might be shortened and some assays substituted or combined to make it both cheaper and faster to perform. Furthermore, the microarray design can be changed to multiplex, e.g. 12-plex as offered by the Roche NimbleGen system, instead of 1-plex as used now to increase the number of samples that can be run per slide. Future data analysis software needs to be customized for routine diagnostic analysis both regarding data handling and easy-to-use. The cost for the assay at the moment is in the hundreds of dollars per sample, but once established and refined as a technique, the cost is estimated to drop significantly but probably never to levels comparable to PCR. However, the benefit with this assay is that it replaces hundreds of PCR reactions with just one reaction, and often hospitals requests more than one PCR assay per patient in the search for a correct diagnose. Furthermore, since the microarray also contains probes for bacteria, it can combine both viral and bacterial diagnostics. Another benefit is that both DNA and RNA viruses can be analysed using the same protocol.

In recent years WGA by MDA has been applied extensively, enabling researchers to get enough material from as little as a single cell for down-stream analysis [13], [32], [33]. Phi29 polymerase amplifies all accessible DNA present in a sample, including any exogenous DNA contamination. For viral or bacterial diagnostic purposes, where presence rather than abundance is important, the main concern is contaminating human gDNA that can compete with pathogen DNA. We show that our pre-treatment can reduce this impact from gDNA and thereby allowing for a greater amplification of the viral DNA. However, in some cases this is not enough due to large amounts of gDNA being present that can not be completely removed and this, combined with low viral copy number, might make certain types of clinical samples less suitable for efficient amplification and microarray analysis. This is currently under investigation. Our microarray analysis of BKV^+^ urine samples indicated that as little as 5 genomic copies of a circular DNA genome could be enough to generate enough material by Phi29-amplification to enable successful identification by the LLMDA. Such a high sensitivity might not be achievable for other types of viral genomes, and is therefore under further investigation. Furthermore, to increase the chance of producing enough material from low copy number samples, we found that the WTA reaction should be allowed to run to completion (8 hours). The sample volume used for purification could also be increased.

Our results show that the protocol established for pre-treatment and amplification followed by microarray analysis is a useful method that potentially could optimize and simplify the current way of doing multiple analyses in a diagnostic laboratory.

## Supporting Information

Table S1 **Representative examples of the effect of DNase-treatment on virus before and after Phi29-amplification.**
(DOC)

Table S2 **The β-actin content in clinical samples, before and after WTA.**
(DOC)
