# Supplementary material for: The Microbial Detection Array Combined with Random Phi29-Amplification Used as a Diagnostic Tool for Virus Detection in Clinical Samples
Source: PLoS One. 2011 Aug 10;6(8):e22631. doi: 10.1371/journal.pone.0022631 (PMC3154197; doi:10.1371/journal.pone.0022631)
Supplement: Table S1 — Representative examples of the effect of DNase-treatment on virus before and after Phi29-amplification. (DOC) [file pone.0022631.s001.doc]

**Table S1. Representative examples of the effect of DNase-treatment on virus before and after Phi29-amplification**

| **Samples** | **HSV1^a^** |  | **HSV1^a^** |  | **HCV^b^** |  |
| --- | --- | --- | --- | --- | --- | --- |
| **Treatment** | **-DNase** | **+DNase** | **-DNase** | **+DNase** | **-DNase** | **+DNase** |
| **Before Phi29^c^** | 18.09 | 19.80 | 22.41 | 26.85 | 27.94 | 29.91 |
| **After Phi29^d^** | 15.92 | 11.97 | 16.85 | 15.10 | 24.80 | 21.56 |
|  |  |  |  |  |  |  |
| **Fold increase^e^** | 112 | 5673 | 1176 | 88 292 | 443 | 8192 |

**Note.** HSV1, Herpes Simplex virus 1; HCV, Hepatitis C virus.

^a^ Two different HSV1-positive clinical skin lesion samples, amplified by GenomiPhi.

^b^ HCV-positive clinical serum sample, amplified by WTA.

^c^ Ct-values from virus-specific real-time PCR before Phi29-amplification.

^d^ Ct-values from virus-specific real-time PCR after Phi29-amplification.

^e^ Fold increase of virus after Phi29-amplification, calculated from ∆Ct-values (before WTA – after WTA) combined with dilution factors for each sample.
