# Supplementary material for: The Microbial Detection Array Combined with Random Phi29-Amplification Used as a Diagnostic Tool for Virus Detection in Clinical Samples
Source: PLoS One. 2011 Aug 10;6(8):e22631. doi: 10.1371/journal.pone.0022631 (PMC3154197; doi:10.1371/journal.pone.0022631)
Supplement: Table S2 — The β-actin content in clinical samples, before and after WTA. (DOC) [file pone.0022631.s002.doc]

**Table S2. The β-actin content in clinical samples, before and after WTA**.

| **Group^a^** | **Virus** | **Sample** | **∆Ct^b^** | **Fold increase β-actin^c^** | **Microarray analysis** |
| --- | --- | --- | --- | --- | --- |
| **dsDNA** | HSV1 | lesion | 31-37 | No amplification | Virus detected |
|  | HPV16 | Cervix | 32-33 | 71 | Virus detected |
|  | BKV | Urine | 37-no Ct | No amplification | Virus detected |
|  | JCV | CSF | No Ct | No amplification | Virus detected |
| **dsRNA** | Rota A | Faeces | 32-21 | 44,000 | Virus detected |
| **(+)ssRNA** | HCV | Serum | No Ct | No amplification | Virus detected |
| **(-)ssRNA** | RSV | TA | 30-26 | 491 | Virus detected |

**NOTE**. WTA, Whole Transciptome Amplification; HSV1, Herpes Simplex virus 1; HPV, Human Papillomavirus; BKV, BK Polyomavirus; JCV, JC Polyomavirus; CSF, cerebrospinal fluid; Rota A, Rotavirus A; HCV, Hepatitis C virus; RSV, Respiratory Syncytial virus; TA, tracheal aspirate; No Ct, undetectable levels.

^a^ Viruses are grouped based on nucleic acid content, according to the Baltimore Classification.

^b^ Difference in Ct-value in β-actin-specific real-time PCR before and after Phi29-amplification.

^c^ Fold increase of β-actin after Phi29-amplification, calculated from ∆Ct combined with dilution factors for each sample.
